# Pearls & Oy-sters: *AARS2* Leukodystrophy—Tremor and Tribulations

**DOI:** 10.1212/WNL.0000000000209296

**Published:** 2024-03-20

**Authors:** Katy Green, Claire L. MacIver, Sian Ebden, D.A. Rees, Kathryn J. Peall

**Affiliations:** From the Cardiff University Brain Research Imaging Centre (CUBRIC) (K.G., C.L.M.), Cardiff University; University Hospital of Wales (S.E.), Cardiff and Vale University Health Board; and Neuroscience and Mental Health Innovation Institute (D.A.R., K.J.P.), Cardiff University, United Kingdom.

## Abstract

A 35-year-old woman with a progressive, bilateral upper limb tremor, personality change, behavioral disturbance, and primary ovarian insufficiency was found to have *AARS2*-related leukodystrophy. She had congenital nystagmus which evolved to head titubation by age 8 years and then developed an upper limb tremor in her mid-teens. These symptoms stabilized during her 20s, but soon after this presentation at age 35 years, neurologic and behavioral disturbances progressed rapidly over a 12-month period requiring transition to an assisted living facility with care support (4 visits/day) and assistance for all activities of daily living. MRI of the brain demonstrated confluent white matter changes predominantly involving the frontal lobes consistent with a leukodystrophy. All other investigations were unremarkable. Nongenetic causes of a leukodystrophy including sexually transmitted diseases and recreational drug use were excluded. Family history was negative for similar symptoms. Gene panel testing identified compound heterozygous pathogenic *AARS2* mutations. This case highlights the importance of MRI brain imaging in progressive tremor syndromes, the utility of gene panels in simultaneous testing of multiple disorders with overlapping phenotypes, and the need for awareness of comorbid endocrinological disorders in many of the genetic leukodystrophies, whose identification may aid in clinical diagnosis.

## Pearls

- Pathogenic *AARS2* variants are a rare cause of leukodystrophy characterized by neurologic, behavioral, and endocrinological phenotypes.
- White matter changes in the form of confluent T2 hyperintensities involving the frontal or periventricular regions on MRI of the brain can differentiate leukodystrophies from other potential causes of progressive movement disorders.

## Oy-sters

- Other causes of leukodystrophies and leukoencephalopathies include recreational drug use and transmissible infective illnesses including sexually transmitted diseases.
- Many genetic leukodystrophies demonstrate autosomal or X-linked recessive patterns of inheritance, and therefore, the absence of a family history should not preclude genetic testing.

## Case Report

A 35-year-old woman presented to movement disorders clinic with a bilateral, asymmetrical upper limb tremor. The tremor began when she was 16 years. It was initially constant in nature but exacerbated by stress. During early adult life, the tremor became more variable in frequency and intensity causing difficulties with writing and eating. She had first been reviewed by neurology at age 4 years following recurrent episodes of dizziness with pendular nystagmus noted on examination. The vertiginous episodes resolved, but the abnormal eye movements persisted. Brain imaging during this period was reported to be within normal limits leading to the diagnosis of benign paroxysmal vertigo with hereditary nystagmus. By age 8 years, she had developed involuntary head movements and an evolution of the eye movements to a vertical nystagmus observed on upward gaze. No further clinical interaction took place until the patient was age 20 years when she presented with ongoing head movements and upper limb tremor.

Medical history included depression, anxiety, ongoing investigations for diabetes, and awaiting specialist input for symptoms consistent with premature ovarian failure. Neurodevelopmentally, she was born at term following a normal pregnancy. There was no reported developmental delay. She attended mainstream school, keeping up with peers academically and during sporting activities. Prescribed medication included sertraline, atorvastatin, and omeprazole. She smoked 10–15 cigarettes/day and had an extensive history of marijuana use.

On initial clinical examination, the patient was alert, orientated, and able to provide the clinical history herself. Speech was spontaneous, and her affect was normal. Neurologic examination was unremarkable with exception of downbeat nystagmus and a bilateral fine upper limb tremor, evident with outstretched arms and in Holme's position. Dystonic posturing was evident in the hands bilaterally (left > right), at rest and with posture, with a reduction in fine motor function and dexterity. No evidence of involuntary head movements was observed during this or subsequent examinations. She was able to walk independently, as well as on heels, toes, and heel-toe walk.

Owing to functional difficulties imposed by the tremor, the patient was started on primidone (100 mg twice daily). Propranolol (160 mg) had been tried previously but had resulted in panic attacks and mood fluctuations. Serial clinical review over the subsequent 2 years found little improvement in tremor with oral medical therapy. There was also progression of the wider clinical symptoms, initially in the form of increasing apathy, changes to behavior (e.g., getting into vehicles with men unknown to her), and verbal and physical aggression in the absence of clear triggers. She developed elements of paranoia, becoming reluctant to take her tablets due to a suspicion of poisoning. A local psychiatrist felt that her symptoms were consistent with low mood and introduced citalopram (40 mg). An antipsychotic was considered but not prescribed due to concerns for increased risk of sedation and falls. There were also changes to language and mobility including a gradual reduction in spontaneous speech limiting her to single word responses to questions and worsening balance including development of a broad-based gait and multiple falls. Progression of symptoms necessitated she move to an assisted living facility with care support 4 times/day for all activities of daily living.

Investigations included serum tests (full blood count, urea and electrolytes, liver function tests, thyroid function tests, calcium, phosphate), causes of progressive disorders (copper, caeruloplasmin, protein electrophoresis, immunoglobulins), and an initial autoimmune screen (ANA, anti-dsDNA, lupus anticoagulant, anti–B2-GP1, anticardiolipin antibodies), all of which were within normal limits. CT head imaging demonstrated evidence of cerebral atrophy (Figure, A), with a contemporaneous noncontrasted MRI of the brain demonstrating frontal predominant T2 hyperintensity (Figure, B), thinning of the corpus callosum on T1 images (Figure, C), and a nonspecifically abnormal MR spectra (Figure, D). These MRI changes suggested a leukodystrophy with potential etiologies including habitual recreational drug use (e.g., heroin and methanol) or infectious diseases such as HIV, syphilis, hepatitis B or C, and tuberculosis. There was no reported history of recreational drug use beyond the marijuana outlined above, and serum testing was negative for infective causes. Therefore, Genomics England adult-onset leukodystrophy gene panel was sent which identified compound heterozygous mutations in the *AARS2* gene (eTable 1, links.lww.com/WNL/D499). Extended family members declined further genetic testing.

**Figure:**
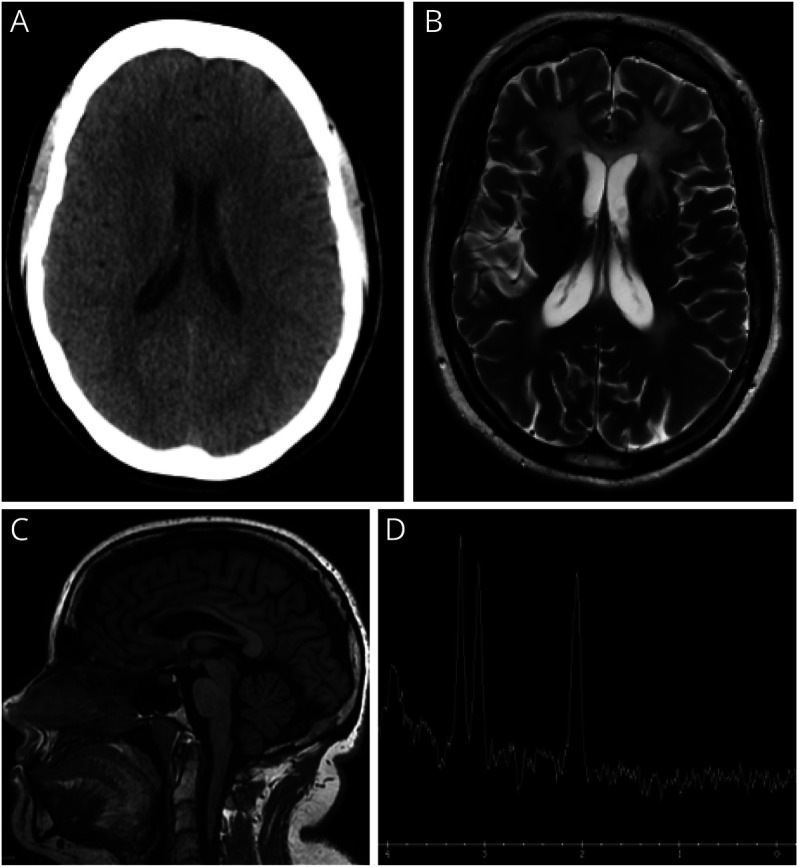
Cross-Sectional Brain Imaging Undertaken at the 6-Year Interval (A) Axial CT head obtained at age 29 years demonstrating no white matter abnormality, but evidence of an interval increase in size of the lateral ventricles consistent with atrophy; (B) axial T2 MRI brain (age 35 years) demonstrating frontal predominant hyperintensity with involvement of the anterior external capsules, anterior limbs of the internal capsule, and splenium of the corpus callosum; (C) sagittal T1 MRI brain (age 35 years) demonstrating a thin corpus callosum secondary to white matter volume loss; and (D) magnetic resonance spectroscopy, single-voxel TW144 ms demonstrating an abnormal Hunter's angle with no lactate peak.

Given that the *AARS2* phenotype also involves primary ovarian failure, coupled with the clinical history of early menopause, the patient was referred to endocrinology for further investigation. Tests included estradiol <55pmol/L (30–400 pmol/L) FSH 24.95 IU/L (4.5–21.5 IU/L), prolactin 217 ng/mL (<25 ng/mL), and LH 15.5IU/L (5–25 IU/L), supporting a diagnosis of premature ovarian failure, with subsequent DEXA scan identifying mild-moderate osteopenia.

## Discussion

The initial symptoms described in this patient included a progressive movement disorder coupled with behavioral change and psychiatric symptoms These symptoms suggest a broad differential diagnosis including Huntington disease, Wilson disease, and anti-NMDA encephalitis, among others. Although the absence of a family history in this case does not align with the autosomal dominant inheritance pattern of Huntington disease, there is overlap in the clinical picture. Specifically, as with this patient, individuals with Huntington disease present with chorea, irritability, and depression while later Parkinsonism and dystonia are frequently observed. By contrast, Wilson disease has an autosomal recessive pattern of inheritance, meaning negative family history would not be uncommon. Typically half of patients present with neurologic signs, 40% with hepatological involvement and only 10% with psychiatric symptoms. Initial symptoms may be mild and nonspecific, such as difficulty concentrating and lack of coordination, with later features including parkinsonism, tremor, and dystonia.^1^ However, abnormalities in serum copper and caeruloplasmin testing would be anticipated. Anti-NMDA encephalitis, with a young (25–35 years) female predominance and linked with ovarian teratomas, shares similar symptoms at presentation. These would include behavioral change and depression, although mania and psychosis are also reported, with later movement disorder emergence and seizures. However, NMDA encephalitis is typically acute or subacute, making it less likely in this patient.^2^ Although not consistent with the clinical phenotype in this case, a wider differential diagnosis for adult-onset movement disorder and psychiatric symptoms includes Parkinson disease, Tourette syndrome, primary dystonia, and the immune mediated anti–leucine-rich glioma-inactivated 1 (LGI1), antiglycine receptor, and anti-D2 dopamine receptor disorders.^3^

In this case, the key investigation was MRI of the brain demonstrating diffuse white matter changes, which raised the possibility of a leukodystrophy. The pattern and anatomical distribution of white matter changes vary with the distinct aetiologies of leukodystrophy but can often aid in guiding next-step investigations, including genetic testing.^4-6^ In the absence of a family history, initial investigations should explore potential acquired causes including testing for HIV, syphilis, hepatitis B/C and tuberculosis, inflammatory disorders such as systemic lupus erythematosus (SLE), and malignancy, particularly primary CNS lymphoma. Other potential causes of a confluent and symmetrical leukoencephalopathy on MRI of the brain include previous chemotherapy (in particular 5-fluorouracil and methotrexate), radiotherapy, and recreational drug use, such as heroin and methanol that would need to be explored during the clinical history.^4,7^

The *AARS2* gene encodes mitochondrial alanyl-tRNA synthetase enzyme which is central in charging of tRNA-ala with alanine during mitochondrial translation.^8^ Loss of this function results in reduction in aminoacylation activity of the synthetase, affecting mitochondrial protein synthesis. Pathogenic *AARS2* mutations are autosomal recessively inherited. The 63 cases reported to date involved a constellation of symptoms including tremor, personality change, dementia, reduced mobility, and leukodystrophy, with progressive symptomatic worsening and reduced functional capacity.^9^ The typical phenotype described is consistent with the case reported here and includes a mean age at presentation of 29 years, initial psychiatric symptoms coupled with cognitive decline, parkinsonism, ataxia, tremor, pyramidal signs, and seizures, followed by severe and rapid motor decline. Nystagmus has been reported in other forms of leukodystrophy, diagnosed in both childhood^10^ and adult life^11^; however, unlike the case reported here, leukodystrophy-associated childhood-onset nystagmus is typically observed in the context of a wider clinical phenotype including global developmental delay and central hypotonia.^10^ Premature ovarian failure is present in almost all female cases. Changes identified on MRI head imaging typically include symmetric and confluent T2 hyperintense/T1 hypointense white matter signal change in the frontoparietal white matter, corpus callosum, and pyramidal tracts.^4^ Only symptomatic management is currently available, with no disease modifying or curative therapy.

More than 50 genes have been implicated in leukodystrophy, with those involving a movement disorder phenotype presented in Table 1.^12^ Ataxia is the most commonly observed movement disorder, with tremor, dystonia, and spasticity also frequently reported. Fewer genetic leukodystrophies are linked with endocrinological abnormalities similar to the primary ovarian failure observed in this case, with these including vanishing white matter disease (VWMD) caused by mutations in the *ElF2B* gene and galactosemia (*GALE*, *GALK1*, and *GALT* gene mutations).^4,13^ Other endocrinological disorders observed in the context of leukodystrophies include adrenal insufficiency (e.g., X-linked adrenoleukodystrophy),^14^ hypogonadism (e.g., VWMD),^15^ growth failure (e.g., POL3 and Cockayne syndrome leukodystrophies),^16^ and hypothyroidism (e.g., Pelizaeus-Merzbacher disease).^17^

**Table:** Genetic Leukodystrophies Involving a Movement Disorder Phenotype

Disorder	Gene (OMIM Number)	Protein & function	Movement disorder phenotype	Wider clinical phenotype
Autosomal Dominant				
Adult-onset autosomal dominant leukodystrophy (ADLD)	*LMNB1* (169500)	*LMNB1* protein maintains the structural integrity of the nucleus, regulating chromatin organization, and influencing gene expression	Spasticity, ataxia, tremor	Autonomic dysfunction (e.g., postural hypotension, bladder and bowel dysfunction), hypertonia, limb weakness, brisk reflexes, nystagmus, dysarthria, dysphagia, later onset cognitive, and psychiatric symptoms
Alexander disease (adult form)	*GFAP* (203450)	*GFAP* is an intermediate filament protein primarily expressed in astrocytes, providing structural support to glial cells, and contributing to the formation and maintenance of the blood-brain barrier	Ataxia, palatal myoclonus	Bulbar or pseudobulbar palsy, spasticity
Hereditary diffuse leukoencephalopathy with spheroids	*CSF1R* (221820)	Encodes a tyrosine kinase growth factor receptor for colony-stimulating factor-1	Parkinsonism, ataxia, spasticity	Dementia, personality change
Autosomal recessive				
Progress Leukoencephalopathy with ovarian failure	*AARS2* (612035)	*AARS2* encodes mitochondrial alanyl-tRNA synthetase involved inn mitochondrial translation	Tremor, ataxia, spasticity, parkinsonism	Cognitive impairment, apathy, seizures, speech impairment which can lead to aphasia, premature ovarian failure
Adult-onset methylenetetrahydrofolate reductase deficiency	*MTHFR* (236250)	*MTHFR* enzyme is involved in folate metabolism, specifically a step in methionine synthesis and DNA methylation	Tremor, ataxia, hypotonia	In adult form, this may present with psychiatric symptoms and neuropathy. Childhood onset forms typically present with hypotonia, feeding problems, developmental delay, microcephaly
Adult-onset metachromatic leukodystrophy	*ARSA* (250100)	*ARSA* enzyme is involved in sulfatide hydrolysis, required for the maintenance of myelin integrity	Spasticity, ataxia, dystonia, chorea	Dementia, apathy, behavioral change, psychosis
Leukoencephalopathy with vanishing white matter (VWM)	VWM1: *EIF2B1* (603896) VWM2: *EIF2B2* (620312) VWM3: *EIF2B3* (620313) VWM4: *EIFB4* (620314) VWM5: *EIFB5* (620315)	*EIF2B* proteins are involved in translation regulation and protein synthesis under stress conditions	Progressive cerebellar ataxia	Spasticity, ovarian failure, cognitive impairment, seizures
Leukoencephalopathy with ataxia	*CLCN2* (615651)	Encodes a voltage-gated chloride channel highly expressed in the adrenal glands	Ataxia	Visual field defects, optic neuropathy, headaches
Krabbe disease	*GALC* (245200)	*GALC* encodes galactosylceramidase, critical for the maintenance of myelin and prevention of accumulation of toxic products	Ataxia	Adult-onset: weakness, visual loss, and cognitive regression Infantile-onset: irritability, spasticity, developmental delay
Galactosemia	GALAC1: *GALT* (230400) GALAC2: *GALK1* (230200) GALAC3: *GALE* (230350) GALAC4: *GALM* (618881)	Enzymes are critical in galactose metabolism	Tremor, dystonia	Feeding difficulties, failure to thrive, jaundice, cataracts, intellectual disability, speech difficulties, developmental delay, hypoglycemia
X-Linked recessive				
Pelizaeus-Merzbacher disease	*PLP1* (312080)	*PLP1* encodes lipophilin the primary constituent of myelin in the CNS	Ataxia, dystonia	Nystagmus, spastic quadriplegia, developmental delay
Adrenoleukodystophy	*ABCD1* (300100)	ABCD1 is an ATP-binding cassette transporter, preventing accumulation of very-long-chain fatty acid	Ataxia, leg weakness, spasticity	Progressive behavioral, neurologic, and cognitive deficits. Bladder and bowel dysfunction, primary adrenocortical insufficiency

## Conclusion

This case adds to the limited literature describing *AARS2* leukodystrophy, highlighting the importance of MRI brain imaging in progressive tremor syndromes, and the awareness of coexistent endocrinological phenotypes in genetic leukodystrophies.

## Appendix Authors

**Table:**

Name	Location	Contribution
Katy Green	Cardiff University Brain Research Imaging Centre (CUBRIC), Cardiff University, United Kingdom	Drafting/revision of the manuscript for content, including medical writing for content; analysis or interpretation of data
Claire L. MacIver, MBBCh, BSc, MSc	Cardiff University Brain Research Imaging Centre (CUBRIC), Cardiff University, United Kingdom	Drafting/revision of the manuscript for content, including medical writing for content; major role in the acquisition of data; analysis or interpretation of data
Sian Ebden, MBBCh, BSc, FRCR	University Hospital of Wales, Cardiff and Vale University Health Board, United Kingdom	Drafting/revision of the manuscript for content, including medical writing for content; analysis or interpretation of data
D.A. Rees, PhD, FRCP	Neuroscience and Mental Health Innovation Institute, Cardiff University, United Kingdom	Drafting/revision of the manuscript for content, including medical writing for content; analysis or interpretation of data
Kathryn J. Peall, MD, PhD, FRCP	Neuroscience and Mental Health Innovation Institute, Cardiff University, United Kingdom	Drafting/revision of the manuscript for content, including medical writing for content; major role in the acquisition of data; study concept or design; analysis or interpretation of data
